# Overexpression of MicroRNA-122 Resists Oxidative Stress-Induced Human Umbilical Vascular Endothelial Cell Injury by Inhibition of p53

**DOI:** 10.1155/2020/9791608

**Published:** 2020-10-27

**Authors:** Hua-qing Li, Zhi-yu Pan, Zhen Yang, Don-bing Zhang, Qian Chen

**Affiliations:** Department of Vascular Surgery, Minhang Hospital, Fudan University, Shanghai, China 201100

## Abstract

Deep venous thrombosis (DVT) constitutes a great threat to health worldwide. Endothelial cell injury and dysfunction comprise the critical contributor for the development of DVT. However, the mechanism behind it remains poorly elucidated. The study is aimed at investigating the role of microRNA-122 (miR-122) and oxidative stress on DVT. The results showed that miR-122 overexpression dampened H_2_O_2_-evoked cytotoxic injury in human umbilical vein endothelial cells (HUVECs) by increasing cell viability, suppressing cell apoptosis and oxidative stress injury. Notably, miR-122 overexpression attenuated provasoconstriction factor endothelin-1 (ET-1) expression in HUVECs exposed to H_2_O_2_ but enhanced the productions of vasodilatation factor Prostaglandin F1*α* (PGF1*α*). Moreover, inhibition of miR-122 had the opposite results. miR-122 could inhibit the expression of p53. Low expression of p53 could enhance the protection of miR-122 on HUVEC injury. This study highlights that miR-122 overexpression may restore H_2_O_2_-induced HUVEC injury by regulating the expression of p53.

## 1. Introduction

Deep vein thrombosis (DVT) affects 1-2 per 1000 people each year. And its prevalence increases with age [1–3]. DVT is associated with a variety of medical conditions including pulmonary or systemic embolism, which is responsible for high mortality rates. In a clinic, the treatments for DVT patients including anticoagulation and surgical intervention are always ineffective. Many DVT patients suffered from postthrombotic syndrome, major bleeding, and even death [4, 5]. Thus, it is essential to study the molecular mechanism of DVT and explore alternative therapies for DVT treatment.

Endothelial cell injury and dysfunction are major factors contributed to DVT. Previous studies reported that excessive oxidative stress is a common cause of vascular endothelial cell injury [6–8]. Moreover, intracellular reactive oxygen species (ROS) can aggravate apoptosis in vascular endothelial cells and decrease antiapoptotic molecule expression [9]. However, the molecular mechanism of oxidation stress-induced endothelial cell injury is unclear.

MicroRNAs are small noncoding RNAs consisting of approximately 22 nucleotides, which have important roles in regulating to target mRNAs for cleavage or translational repression. Many microRNAs affect cell proliferation and apoptosis, which are critical to the development and progression of vascular disease. MicroRNA-122 (miR-122), a liver-specific miRNA, is involved in regulating lipid metabolism, iron homeostasis, and differentiation of hepatocytes. A previous study demonstrated that miR-122 was induced by ROS in human endothelial cells and its expression is associated with endothelial cell apoptosis [10]. Besides, the tumor suppressor p53 was a potential target gene of miR-122. Accumulating evidence suggests an important function for p53 in promoting cell apoptosis [11, 12]. Of note, endothelial injury is alleviated by ROS through the induction of endothelial cell apoptosis. However, the regulatory relationship among miR-122, p53, and oxidation stress-induced vascular endothelial cell injury is not completely understood. The present study was conducted to study the role of miR-122 in vascular endothelial cell injury. Furthermore, we investigate whether miR-122 regulates p53 participating in oxidation stress-induced vascular endothelial cell apoptosis.

## 2. Materials and Methods

### 2.1. Clinical Samples

The study was approved by the Human Ethics Committee Review Board at Minhang Hospital, and informed consent was obtained from all patients. A total of 30 blood samples were obtained from patients (age range, 45–80 years; sex ratio, 1 : 1) that were diagnosed with DVT at Minhang Hospital between 2018 and 2019. In addition, 30 blood samples were obtained from healthy controls (age range, 45–80 years; sex ratio, 1 : 1). Blood samples were used to detect miRNA-122.

### 2.2. Establishment of the Human Umbilical Vein Endothelial Cell (HUVEC) Oxidative Stress Injury Model Induced by H_2_O_2_

HUVECs were purchased from the Chinese Academy of Medical Sciences and Peking Union Medical College (Beijing, China). HUVECs were grown in Dulbecco's Modified Eagle's Medium (DMEM) containing 10% fetal bovine serum (FBS). To establish the oxidative stress injury model, cells were exposed to H_2_O_2_ (0.5 mM) for 24 h. All cells were incubated in an incubator of 5% CO_2_ at 37°C.

HUVECs treated with H_2_O_2_ (0.5 mM) for 24 h were used as the H_2_O_2_ group. HUVECs treated with H_2_O_2_ (0.5 mM) transfected with the negative control (NC) sequence of miR-122 analogs were used as H_2_O_2_+mimic-NC. HUVECs treated with H_2_O_2_ (0.5 mM) transfected with the sequence of miR-122 analogs were used as H_2_O_2_+mimic-122. HUVECs treated with H_2_O_2_ (0.5 mM) transfected with the NC sequence of miR-122 inhibitor were used as H_2_O_2_+inhibitor-NC. HUVECs treated with H_2_O_2_ (0.5 mM) transfected with the sequence of miR-122 inhibitor were used as H_2_O_2_+inhibitor-122. HUVECs treated with H_2_O_2_ (0.5 mM) transfected with the NC sequence of miR-122 inhibitor and the NC sequence of p53 siRNA were used as H_2_O_2_+inhibitor-NC+siRNA-NC. HUVECs treated with H_2_O_2_ (0.5 mM) transfected with the sequence of miR-122 inhibitor and the NC sequence of p53 siRNA were used as H_2_O_2_+inhibitor-122+siRNA-NC. HUVECs treated with H_2_O_2_ (0.5 mM) transfected with the sequences of miR-122 inhibitor and p53 siRNA were used as H_2_O_2_+inhibitor-122+siRNA-122.

### 2.3. CCK-8

HUVECs were seeded into a 96-well plate at a density of 6∗10^3^ cells/well and incubated for 48 h. After that, 10 *μ*l CCK-8 reagent (Cell Counting Kit-8; KeyGen, Nanjing, China) was added to each well. The well plate was grown in a CO_2_ incubator (5% CO_2_) at 37°C for 2 h, and the optical density (OD) was measured at a wavelength of 450 nm. Cell viability was calculated as follows: cell viability (%) = [(experimental well OD450 value − blank well OD450 value)/(control well OD450 value − blank well OD450 value)]∗100.

### 2.4. Apoptosis Detection by Flow Cytometry

HUVECs were seeded into 6-well plates, and the assay was conducted according to the manufacturer's protocol of the apoptosis detection kit (Nanjing KeyGen Biotech Co., Ltd.). Briefly, cells adhering to the wall were digested with 0.25% trypsin and then washed with PBS for three times. The collected cells were detected using flow cytometry (BD FACSArial I; BD Biosciences). The percentage of apoptotic cells in each quadrant was calculated using the FlowJo software (version 7.2.2; FlowJo).

### 2.5. Measure of ROS, Malonaldehyde (MDA), and Superoxide Dismutase (SOD) Content

For ROS measurement, the 2′,7′-dichlorodihydrofluorescein diacetate (DCFH-DA) (Sigma) was used. Briefly, cells were washed three times with PBS and stained with 20 *μ*M of DCFH-DA for 30 min. The fluorescence intensity of samples was tested by a microplate fluorometer (Molecular Devices Corporation, Sunnyvale, CA, USA) using excitation at 480 nm and emission at 530 nm.

For the MDA test, the commercial MDA detection kit (Nanjing Jiancheng Bioengineering Institute, Nanjing, China) was used. The reaction was conducted based on the instruction of the manufacturer. The absorbance of 532 nm was determined.

The content of SOD was detected to assess the antioxidant status in accordance with the recommendation of a commercial SOD assay kit (Randox, Crumlin, UK). All protocols were performed according to the instruction of the manufacturer.

### 2.6. Endothelin-1 (ET-1) and Prostaglandin F1*α* (PGF1*α*) Measurements

ET-1 and PGF1*α* in HUVECs were measured using an enzyme-linked immunosorbent assay (ELISA) kit (Sigma-Aldrich, MO, USA) according to the manufacturer's instructions.

### 2.7. Double-Luciferase Reporter Gene Assay

The target gene statistics of miR-122 were carried out by using the database TargetScan. p53 was selected as the direct target gene of miR-122. The total length of the 3′UTR region of the wild-type (wt) p53 gene was cloned and amplified. The p53-mutant type (mut) vector was constructed by a site-directed mutation of the binding site between miR-122 and the target gene, which was predicted by bioinformatics information. The gene vectors (wt-p53 and mut-p53) and miR-122 mimic were cotransfected into HUVECs to detect the activity of double luciferase according to the method provided by Promega Corporation (Madison, WI).

### 2.8. RNA Extraction and Quantification

TRIzol reagent was used to extract total RNA from serum samples and HUVECs. A total of 1 *μ*g RNA was subjected to reverse transcription using the PrimeScript RT Reagent Kit (Takara, Shiga, Japan). Gene expression was evaluated by quantitative reverse transcription PCR (qPCR) analysis using SYBR Green reagents (SYBR Premix Ex Taq) and the LightCycler Real-Time PCR System (Roche Diagnostics, Basel, Switzerland). qPCR reactions were conducted in a final volume of 10 *μ*l. The threshold cycle (Ct) value was computed for each amplification curve. The results were expressed as fold change compared to control values using the 2^-*ΔΔ*CT^ formula.

### 2.9. Knockdown of p53 Expression by Its Specific siRNA

HUVECs were transfected with nontargeting siRNA (siRNA-NC) or p53-targeting siRNA (p53 siRNA) (GeneChem, Shanghai, China) using Lipofectamine 2000 (Invitrogen) following the manufacturer's instructions (Life Technologies, Carlsbad, CA, USA). The knockdown efficiency of p53 targeting siRNA was evaluated by qPCR and western blotting 72 h posttransfection.

### 2.10. Protein Preparation and Western Blotting Analysis

Total protein was extracted using RIPA buffer. The protein specimens (40 mg per lane) were separated using 10% SDS-PAGE and subsequently electroblotted onto a PVDF membrane (Millipore, Billerica, MA, USA). The first antibodies were incubated at 4°C overnight, and the second antibodies were incubated at room temperature for 1 h. Then, the ECL reagent (Amersham Pharmacia, Piscataway, NJ, USA) was performed to visualize the binding signals. Band intensity was quantified by the ImageJ software.

### 2.11. Statistical Analysis

All experiments were repeated at least three times. All data were analyzed using the GraphPad Prism software (GraphPad Software Inc., CA, USA) and expressed as the mean ± standard error (SE). One-way ANOVA and two-way ANOVA were used to compare differences among multiple groups, and the nonpaired *t* test was used to analyze two groups after homogeneity of variance testing. We considered differences to be statistically significant at *p* < 0.05.

## 3. Result

### 3.1. Serum Levels of miR-122 Were Low in Patients with DVT

**Figure 1:**
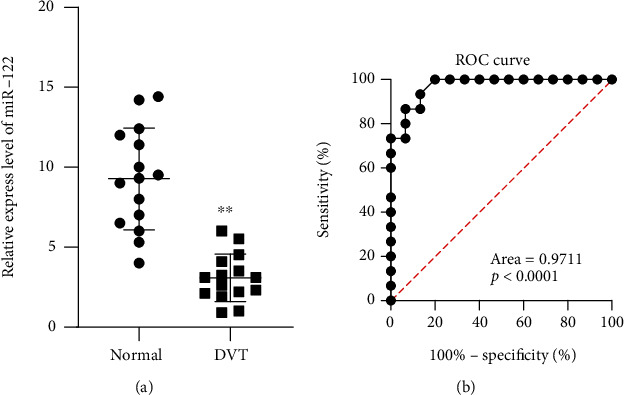
Serum levels of miR-122 in patients with DVT. (a) Serum miR-122 level in the DVT patients and controls by qPCR. (b) ROC curve analysis was conducted. ^∗∗^*p* < 0.01 versus control (*t* test). *n* = 30 per group. All data represent means ± standard error (SE).

Serum miR-122 was detected in the DVT patients and healthy controls using qPCR and then expressed as relative expression compared to the external reference. The expression of miR-122 in serum was ~2 fold lower in the DVT patients than that in the normal controls (Figure 1(a)). We next performed receiver operating characteristic (ROC) curve analysis to evaluate the diagnostic power of miR-122 to distinguish between DVT patients and healthy controls. Here, we found a great diagnostic potential for miR-122, which showed an AUC of 0.9711 (Figure 1(b)).

### 3.2. The Cytotoxic Effects of H_2_O_2_ on HUVECs

**Figure 2:**
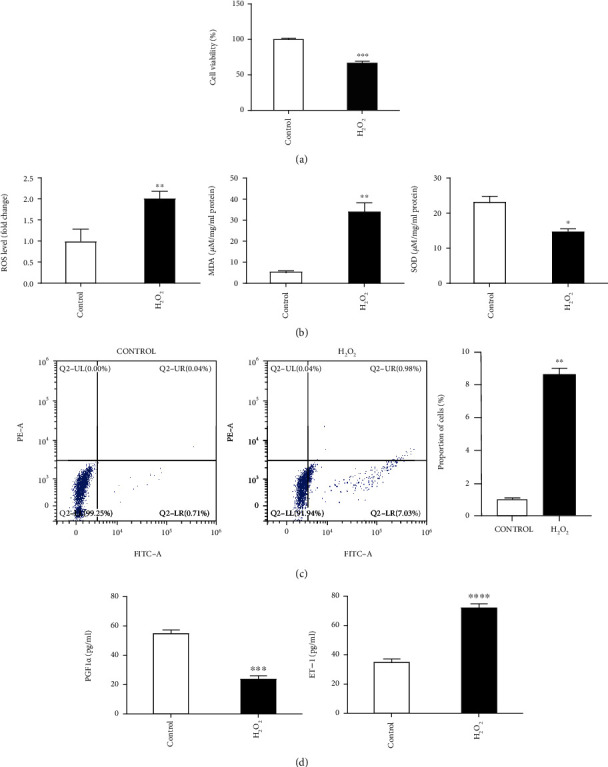
H_2_O_2_ treatment induced HUVEC injury. (a) Effect of H_2_O_2_ treatment on the proliferation of HUVECs by CCK-8 assay. (b) Expression of oxidative stress-related factors including ROS, MOD, and SOD in HUVECs treated by H_2_O_2_. (c) Analysis of apoptosis rate in H_2_O_2_-treated HUVECs by flow cytometry. (d) Effect of H_2_O_2_ exposure on thrombosis-related factor (PGF1*α* and ET-1) expression in HUVECs. ^∗^*p* < 0.05, ^∗∗^*p* < 0.01, ^∗∗∗^*p* < 0.001, and ^∗∗∗∗^*p* < 0.0001 versus control (*t* test). Data are representative of at least three different experiments. All data represent means ± standard error (SE).

To assess the effects of H_2_O_2_ exposure on HUVECs, we firstly tested cell proliferation of HUVECs using the CCK-8 assay (Figure 2(a)). The results showed that the cell viability was significantly lower following treatment with H_2_O_2_ compared with control. Meanwhile, the results of oxidative stress detection demonstrated that compared with control HUVECs, the levels of ROS and MDA in the H_2_O_2_-treated cells increased ~1 fold and ~5 fold, respectively, while that of SOD was decreased by ~35% than the control group (Figure 2(b)). Besides, the cell apoptosis rate was detected by flow cytometry. And the results demonstrated that the H_2_O_2_ treatment remarkably promoted HUVEC apoptosis, which is ~9 fold to that of the control group (Figure 2(c)). These results suggested that H_2_O_2_ treatment induced HUVEC injury.

To our knowledge, endothelial cell injury plays a critical role in thrombosis; we therefore further investigated the effect of H_2_O_2_ exposure on thrombosis-related factor expression in HUVECs. The results indicated that H_2_O_2_ stimulation markedly decreased vasodilatation-related factor contents of PGF1*α*, a stable metabolite of PGI2. Meanwhile, H_2_O_2_ exposure also induced a significant increase in transcripts of ET-1, a critical regulator for vasoconstriction (Figure 2(d)). These results indicated that H_2_O_2_ exposure to HUVECs may promote thrombosis.

### 3.3. Targeted Inhibition of p53 Expression by miR-122

**Figure 3:**
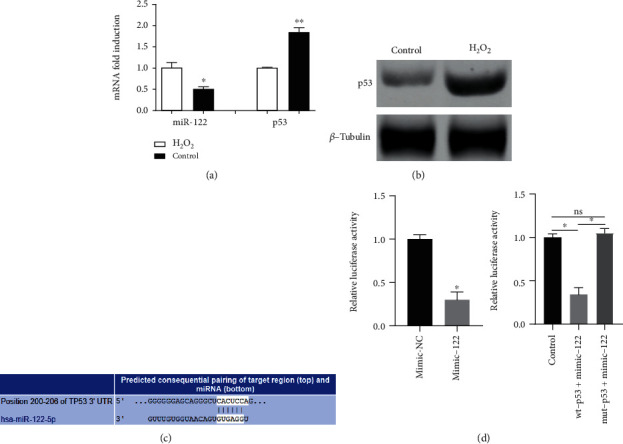
Interaction between miR-122 and p53. (a) Detection of miR-122 and p53 mRNA expression in H_2_O_2_-treated HUVECs by qPCR. (b) Protein bands of p53 in H_2_O_2_-treated HUVECs. (c) TargetScan database predicts that p53 is the target gene of miR-122. (d) Luciferase activity in each group by double-luciferase activity determination. ^∗^*p* < 0.05 and ^∗∗^*p* < 0.01 versus control or mimic-NC (*t* test). Data are representative of at least three different experiments. All data represent means ± standard error (SE).

The results of qPCR and western blot analysis showed that, in comparison to the control HUVECs cells, the miR-122 expression showed an evident decrease and the expression of p53 mRNA and protein was consistently increased in the H_2_O_2_-treated cells (Figures 3(a) and 3(b)). By searching the TargetScan database, we found that p53 was a potential target gene of miR-122 (Figure 3(c)). The results of the double-luciferase reporter gene assay indicated that the luciferase activity of p53-wt in the miR-122 mimic group was significantly lower than that of the miR-122 NC group (Figure 3(d)). No significant difference was found in the luciferase activity of p53-mut in the miR-122 mimic group (Figure 3(d)). The results suggested that miR-122 could inhibit the expression of p53.

### 3.4. miR-122 Reversed H_2_O_2_-Induced HUVEC Injury

**Figure 4:**
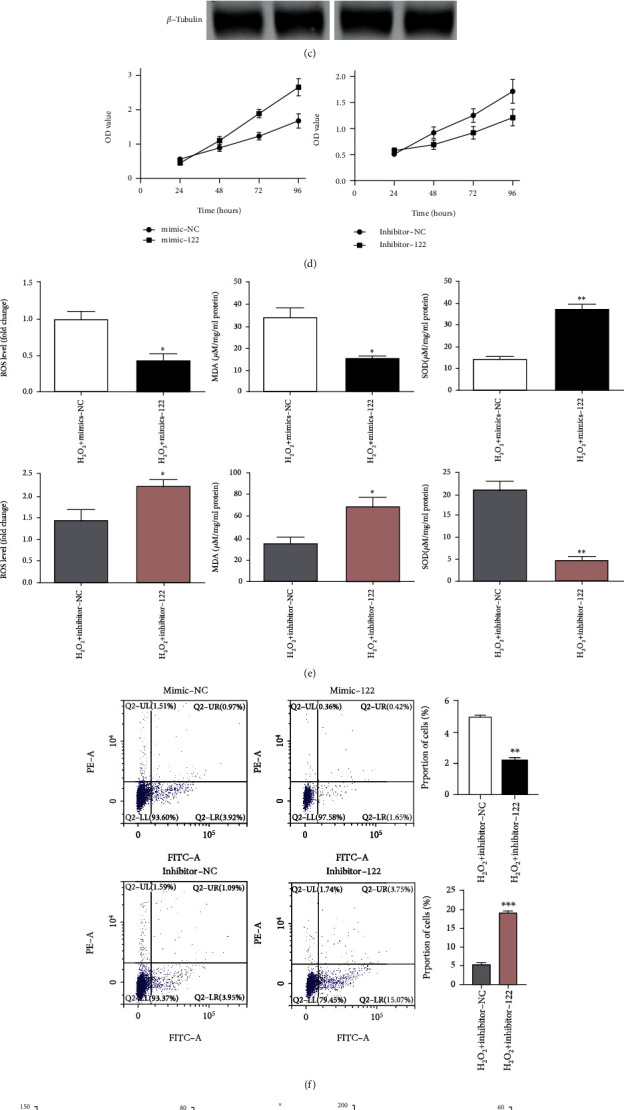
miR-122 reversed H_2_O_2_-induced HUVEC injury. (a) Detection of miR-122 and p53 mRNA expression in HUVECs of the H_2_O_2_+mimic-122 group and the H_2_O_2_+mimic-NC group by qPCR. (b) Detection of miR-122 and p53 mRNA expression in HUVECs of the H_2_O_2_+inhibitor-122 group and the H_2_O_2_+inhibitor-NC group by qPCR. (c) Protein bands of p53 in HUVECs of the H_2_O_2_+mimic-122 group, the H_2_O_2_+mimic-NC group, the H_2_O_2_+inhibitor-122 group, and the H_2_O_2_+inhibitor-NC group by western blotting analysis. (d) Detection of cell survival rate of HUVECs in the H_2_O_2_+mimic-122 group, the H_2_O_2_+mimic-NC group, the H_2_O_2_+inhibitor-122 group, and the H_2_O_2_+inhibitor-NC group by the CCK-8 assay. (e) Expression of oxidative stress-related factors in HUVECs of the H_2_O_2_+mimic-122 group, the H_2_O_2_+mimic-NC group, the H_2_O_2_+inhibitor-122 group, and the H_2_O_2_+inhibitor-NC group. (f) Analysis of apoptosis rate in HUVECs of the H_2_O_2_+mimic-122 group, the H_2_O_2_+mimic-NC group, the H_2_O_2_+inhibitor-122 group, and the H_2_O_2_+inhibitor-NC group by flow cytometry. (g) Thrombosis-related factor (ET-1 and PGF1*α*) expression in HUVECs of the H_2_O_2_+mimic-122 group, the H_2_O_2_+mimic-NC group, the H_2_O_2_+inhibitor-122 group, and the H_2_O_2_+inhibitor-NC group. ^∗^*p* < 0.05, ^∗∗^*p* < 0.01, and ^∗∗∗^*p* < 0.001 versus the H_2_O_2_+mimic-NC group or the H_2_O_2_+inhibitor-NC group (*t* test). Data are representative of at least three different experiments. All data represent means ± standard error (SE).

The results of qPCR and western blot analysis showed that the expression of miR-122 was increased significantly while p53 mRNA and protein expression was both distinctly decreased in the H_2_O_2_+mimic-122 group, as compared with the H_2_O_2_+mimic-NC group (Figures 4(a) and 4(c)). Furthermore, the H_2_O_2_+inhibitor-122 group had a much lower miR-122 expression level and an increment in the expression of p53 mRNA and protein (Figures 4(b) and 4(c)). Besides, the time-dependent cell proliferation curves via the CCK-8 assay indicated that the cell viability in the H_2_O_2_+mimic-122 group was significantly increased throughout 96 h compared with the H_2_O_2_+mimic-NC group (Figure 4(d)), but it was markedly decreased in the H_2_O_2_+inhibitor-122 group compared with H_2_O_2_+inhibitor-NC (Figure 4(d)). As for the oxidative stress detection, the H_2_O_2_+mimic-122 group showed much lower ROS and MDA levels as well as a markedly increased SOD level when compared with the H_2_O_2_+mimic-NC groups (Figure 4(e)). In contrast, the levels of ROS and MDA were increased and the SOD level was decreased significantly in the H_2_O_2_+inhibitor-122 group relative to H_2_O_2_+inhibitor-NC (Figure 4(e)). Furthermore, the flow cytometry results indicated that the apoptosis rate of the H_2_O_2_+mimic-122 group was ~55% lower than that of the H_2_O_2_+mimic-NC group (Figure 4(f)), while the opposite results were found in the H_2_O_2_+inhibitor-122 group (Figure 4(f)). Moreover, we investigated the effect of miR-122 on the expression of thrombosis-related factors in HUVECs. The data showed that in contrast to the H_2_O_2_+mimic-NC group, the significantly lower level of ET-1 and the higher level of PGF1*α* were shown in the H_2_O_2_+mimic-122 group (Figure 4(g)). Simultaneously, the opposite results were shown in the H_2_O_2_+inhibitor-122 group (Figure 4(g)). These results showed that the enhancement of miR-122 expression in H_2_O_2_-induced HUVEC injury could enhance cell activity, inhibit oxidative stress, resist cell injury, and thus reverse thrombosis.

### 3.5. Knockdown of p53 Enhanced the Positive Effect of miR-122 on H_2_O_2_-Induced HUVEC Injury

**Figure 5:**
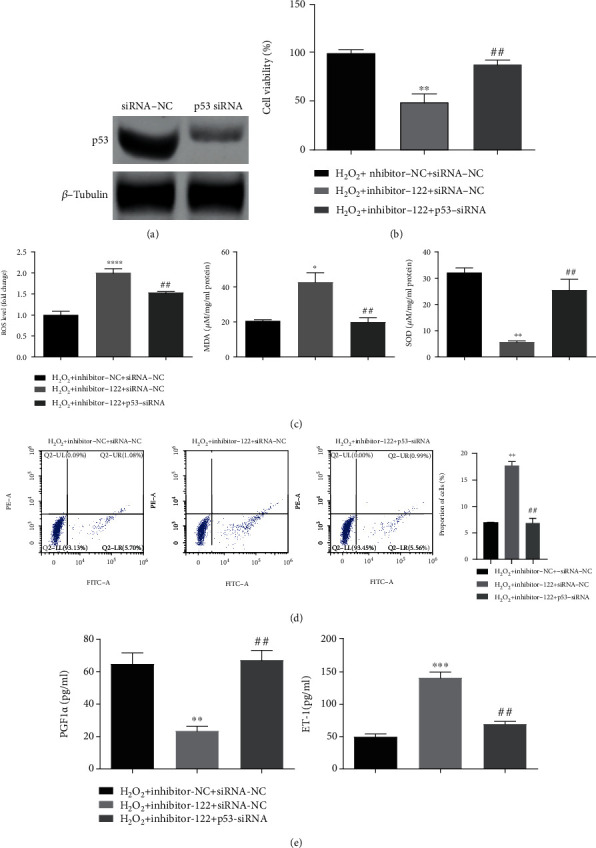
Knockdown of p53 enhanced the positive effect of miR-122 on HUVEC injury. (a) The p53 protein expression in the siRNA-NC group and the p53 siRNA group by western blotting. (b) Detection of cell survival rate of HUVECs in the H_2_O_2_+inhibitor-122+siRNA-p53 group, the H_2_O_2_+inhibitor-122+siRNA-NC group, and the H_2_O_2_+inhibitor-NC+siRNA-NC group. (c) Expression of oxidative stress-related factors in the H_2_O_2_+inhibitor-122+siRNA-p53 group, the H_2_O_2_+inhibitor-122+siRNA-NC group, and the H_2_O_2_+inhibitor-NC+siRNA-NC group. (d) Analysis of apoptosis rate in HUVECs of the H_2_O_2_+inhibitor-122+siRNA-p53 group, the H_2_O_2_+inhibitor-122+siRNA-NC group, and the H_2_O_2_+inhibitor-NC+siRNA-NC group by flow cytometry. (e) Thrombosis-related factor (PGF1*α* and ET-1) expression in HUVECs of the H_2_O_2_+inhibitor-122+siRNA-p53 group, the H_2_O_2_+inhibitor-122+siRNA-NC group, and the H_2_O_2_+inhibitor-NC+siRNA-NC group. ^∗^*p* < 0.05, ^∗∗^*p* < 0.01, and ^∗∗∗^*p* < 0.001 represent the H_2_O_2_+inhibitor-122+siRNA-NC group versus the H_2_O_2_+inhibitor-NC+siRNA-NC group; ^##^*p* < 0.01 represent the H_2_O_2_+inhibitor-122+siRNA-p53 group versus the H_2_O_2_+inhibitor-NC+siRNA-NC group (one-way ANOVA). Data are representative of at least three different experiments. All data represent means ± standard error (SE).

In comparison with the siRNA-NC group, the expression of p53 protein was significantly decreased in the p53 siRNA group, suggesting the successful knockdown of p53 in HUVECs (Figure 5(a)). Besides, in the H_2_O_2_+inhibitor-122+siRNA-NC group, the cell survival rate was decreased evidently (Figure 5(b)), the levels of ROS and MDA were increased while that of SOD was decreased (Figure 5(c)), the apoptosis rate was increased (Figure 5(d)), the PGF1*α* expression was downregulated, and the ET-1 expression was upregulated (Figure 5(e)). In contrast, the H_2_O_2_+inhibitor-122+siRNA-p53 group had the opposite change in each index, which almost restored the effects produced by the H_2_O_2_+inhibitor-122+siRNA-NC group (Figures 5(b)–5(e)). It was suggested that the downregulation of p53 could enhance the protective effect of miR-122 on HUVEC injury induced by H_2_O_2_.

## 4. Discussion

In the present study, we identified the role of miR-122 in DVT and revealed its regulatory functions and molecular mechanisms in HUVEC injury. The results demonstrated that the expression of miR-122 was significantly lower in the DVT patients compared to the normal controls. Moreover, miR-122 had a great diagnostic potential for DVT. Besides, miR-122 overexpression attenuated H_2_O_2_-induced endothelial cell injury through inhibiting the expression of p53. Simultaneously, miR-122 also suppressed ET-1 expression but increased the PGF1*α* level in H_2_O_2_-stimulated HUVECs. The findings of this study suggested that miR-122 may resist HUVEC injury and then support a potential approach against DVT.

The integrity of the vascular endothelial cell is critical to maintain blood vessel function and nonthrombotic state. Endothelial cell injury under oxidative stress is the key regulator in the development of DVT by evoking prothrombosis response [13–16]. In the study, we confirmed that H_2_O_2_ induced vascular endothelial cell oxidative stress injury by increasing ROS and MDA accumulation and decreasing antioxidant enzymatic SOD level. H_2_O_2_ can cause oxidative damage because it can be converted into hydroxyl radicals and oxygen radicals in liver cells [17]. Oxidative stress can activate apoptosis-related signaling pathways in vascular endothelial cells, which then lead to endothelial dysfunction. It is known that endothelial dysfunction is critical to contribute to the initiation of thrombotic diseases including DVT [18]. Moreover, it is reported that patients with DVT had increased oxidative stress levels compared with healthy volunteers in a clinic [16]. In accordance with these reports, we also found that H_2_O_2_ stimulation induced cell apoptosis and then promoted provasoconstriction ET-1 expression and suppressed provasodilatation PGF1*α* release in HUVECs.

In the present study, we found that oxidative stress induced by H_2_O_2_ inhibited miR-122 expression in HUVECs. miR-122 overexpression antagonized H_2_O_2_-triggered oxidative stress injury by lowering the expression of ROS and MDA and increasing SOD levels. Furthermore, miR-122 overexpression attenuated H_2_O_2_-inhibited cell viability and improved cell apoptosis. Similarly, inhibition of miR-122 aggravated H_2_O_2_-induced oxidative stress injury and cell apoptosis in HUVECs. These results indicated that miR-122 had positive roles in ameliorating oxidative stress-induced endothelial cell injury. Intriguingly, amounts of studies reported that attenuating oxidative stress damage and apoptosis in vascular endothelial cells inhibits the development of DVT [19–21]. Altogether, it is reasonable to believe that the enhancement of miR-122 expression represses the development of thrombosis. Moreover, our findings further support it. The results showed that miR-122 affected the expression of thrombosis-related factors. Particularly, miR-122 overexpression attenuated provasoconstriction factor endothelin-1 (ET-1) expression and enhanced the productions of vasodilatation factor Prostaglandin F1*α* (PGF1*α*). In recent years, miR-122 has been reported to play a vital role in hepatocellular carcinoma, cardiomyocyte injury, and insulin resistance [22–25]. Additionally, previous studies reported that miR-122 was associated with vascular injury and oxidative stress [26, 27]. Here, we revealed the new function of miR-122 in HUVEC injury and thrombosis.

Also, our study proposed that miR-122 could inhibit the expression of p53. A previous study showed that miR-122 overexpression decreased cell apoptosis in cutaneous T-cell lymphoma via inhibition of p53 [28]. For the purpose of determining the target gene that miR-122 regulated in HUVEC injury, we analyzed the expression of p53 and found that miR-122 could inhibit the expression of p53. It is indicated that p53 is a target gene of miR-122 in HUVECs. Besides, knockdown of p53 promoted the inhibitory effect of miR-122 on oxidative stress, cell apoptosis, and thrombosis-related factor expression.

In conclusion, the present study revealed that miR-122 overexpression in H_2_O_2_-induced HUVEC injury could inhibit oxidative stress and cell apoptosis, enhance cell activity, and thus resist cell injury. Furthermore, miR-122 overexpression suppressed the expression of thrombosis-related factors. Importantly, low expression of p53 could enhance the protective effect of miR-122 on HUVEC injury. Therefore, these findings indicated that miR-122 may attenuate the pathogenic progression of DVT by ameliorating endothelial cell injury and subsequent prothrombotic response, which provided a potential strategy for DVT.

## Data Availability

The data used to support the findings of this study are included within the article.

## Additional Points

*Highlights*. (1) The expression of miR-122 in serum was significantly low in the DVT patients, and miR-122 has great potential to diagnose DVT. (2) miR-122 overexpression dampened H_2_O_2_-evoked cytotoxic injury in human umbilical vein endothelial cells (HUVECs) by increasing cell viability, suppressing cell apoptosis and oxidative stress injury. (3) miR-122 overexpression attenuated provasoconstriction factor endothelin-1 (ET-1) expression in HUVECs exposed to H_2_O_2_ but enhanced the productions of vasodilatation factor Prostaglandin F1*α* (PGF1*α*). (4) miR-122 could inhibit the expression of p53. (5) miR-122 overexpression may restore H_2_O_2_-induced HUVEC injury by regulating the expression of p53.
